# Antibacterial effects of silver and titanium dioxide nanoparticles-coated orthodontic arch wires: in vitro study

**DOI:** 10.1186/s12903-026-08996-y

**Published:** 2026-06-29

**Authors:** Amany M. Elsonny, Nehal F. Albelasy, Marwa S. Shamaa, Shaza M. Hammad

**Affiliations:** Orthodontic Department, Faculty of Dentistry, Mansoura University, Mansoura, Egypt

**Keywords:** Orthodontic arch wires, Titanium dioxide nanoparticles, Silver nanoparticles, Streptococcus mutans

## Abstract

**Background:**

This study aimed to evaluate the antibacterial efficacy of silver and titanium dioxide nanoparticle coatings applied to stainless steel and nickel-titanium orthodontic arch wires against the Streptococcus mutans bacterium.

**Methods:**

A total of 198 arch wires were divided into two groups based on the type of arch wire material: 99 stainless steel (SS) wires and 99 nickel titanium (NiTi) wires. Every group of these wire materials was evenly subdivided into three subgroups according to the nanocoating material: non-coated group (control), silver nanoparticles (Ag-NPs) and titanium dioxide nanoparticles (TiO_2_NPs). The antibacterial effect was then assessed using the colony-forming unit (CFU), minimum inhibitory concentration (MIC), and agar diffusion technique “minimum zone of inhibition” against S. mutans. The sample included arch wire lengths of 5 millimeters for CFU-MIC tests and 10 millimeters for agar diffusion tests. The collected data were compared using a two-way ANOVA and the Bonferroni test was applied for multiple pairwise comparisons. In the CFU test all samples had been incubated in 10 milliliters of artificial saliva, and the data were collected at intervals of immediately(baseline), after one week, and after two weeks, respectively, then compared using a three-way mixed ANOVA.

**Results:**

The nano-coating effect on arch wires coated with Ag-NPs and TiO_2_NPs demonstrated a strong protective effect against S. mutans (*p* < .001) compared to uncoated wires in the agar diffusion test and in the CFU test. Ag-NP-coated arch wires demonstrated the highest antibacterial activity across all tests. SS arch wires showed significantly greater antibacterial efficacy than NiTi wires (*p* = .008) in the agar diffusion test. Although antibacterial activity decreased over time, Ag-NP-coated wires maintained a detectable effect after one week, whereas after two weeks, the activity of all coatings was markedly reduced.

**Conclusions:**

Coatings of Ag-NPs and TiO_2_NPs significantly enhance antibacterial effect against *S. mutans in vitro*, which significantly contribute to reducing the incidence of dental caries, particularly in SS arch wires coated with Ag-NPs. Although the antibacterial activity decreased over time, this reduction may be associated with potential nanocoating degradation.

## Background

Fixed orthodontic appliances create stagnant areas that facilitate microbial accumulation, increasing the risk of gingivitis, periodontal disease and dental caries [1]. A primary concern during therapy is the development of white spot lesions (WSLs) which is the enamel demineralization resulting from prolonged plaque retention around brackets and arch wires providing orthodontic patients at high risk of developing enamel WSLs leading to unfavorable aesthetic alterations [2, 3]. Streptococcus mutans (S. mutans) is the principal pathogen in this process due to its acidogenic nature and ability to form resilient biofilms so, controlling S. mutans’ levels and activity is therefore essential for the prevention and management of WSLs and the maintenance of enamel integrity [4]. Consequently, modifying the surface of orthodontic components to inhibit bacterial adhesion is a critical strategy for maintaining enamel integrity [5].

Orthodontic arch wires, particularly stainless steel (SS) and nickel-titanium (NiTi) alloys, play a central role in fixed appliance therapy and are continuously exposed to the oral environment. NiTi wires are flexible and necessary for leveling and alignment that are employed in the early stages of treatment [6]. SS arch wires are utilized as the main working arches during the majority of orthodontic treatment because of their mechanical qualities including rigidity, resilience, formability, frictional resistance in addition to their biocompatibility, environmental durability and affordability [6, 7].

The use of nanotechnology in orthodontic appliances is currently one of the most significant developments in the world of dental materials [8] Nanoparticles (NPs) which are insoluble substances with a diameter of less than 100 nanometers are very effective in preventing bacterial adhesion and plaque accumulation and because of their reduced size, NPs are thought to be able to effectively exert their antibacterial characteristics by penetrating the bacterial cell wall [8, 9]. It has recently been shown that silver NPs (Ag-NPs) have remarkable antibacterial capabilities against a variety of microbes as S. mutans due to the release of Ag ions, which can be created and introduced by the oxidative disintegration of Ag-NPs in the presence of oxygen [10–12]. Recently, significant focus has been directed towards titanium dioxide metal NPs (TiO_2_NPs) due to their low toxicity and photocatalytic properties as TiO_2_NPs exhibit antibacterial properties through the generation of hydroxyl radicals, which, upon exposure to ultraviolet light, impede the proliferation of microbes in aqueous solutions [13, 14]. Coating techniques come in a wide variety, including radiofrequency magnetron sputtering, thermal evaporation, physical vapor deposition and the most common sol-gel thin film coating approach which was applied in this investigation [15]. Sol-gel coating technique benefits include high material purity, good structural homogeneity and the ability to produce stable and uniform thin-film coatings [16].

Therefore, the present study aimed to evaluate and compare the antibacterial efficacy of Ag-NPs and TiO_2_NPs coated on SS and NiTi orthodontic arch wires against S. mutans using multiple microbiological tests of agar diffusion, MIC and CFU across different time dependent assays. The null hypothesis of this study proposed that coating SS and NiTi orthodontic arch wires with Ag-NPs and TiO_2_NPs would not significantly enhance the antibacterial activity.

## Materials and methods

### Study design and ethical approval

This in vitro experimental study was approved by the dental research ethics committee of the Faculty of Dentistry at Mansoura University in Egypt (code no. A01303024 OR).

### Collection and distribution of the dataset

A total of 198 SS and NiTi arch wires 0.017 × 0.025 inches (Orthometric, Marília, Brazil) measured 5 millimeters for the colony forming unit (CFU) and minimum inhibitory concentration (MIC) tests and 10 millimeters for agar diffusion test were used in this study. Ag-NPs and TiO_2_NPs were prepared at the National Research Center’s laser research unit in Cairo. A standard S. mutans strain (ATCC 25175) was produced at Ain Shams university’s Faculty of Agriculture’s Central Food Safety Lab. Artificial saliva was made at Mansoura University’s Faculty of Pharmacy’s Chemical Lab which was used for incubation in CFU test.

### Sample size calculation

Sample size was calculated by using Power Analysis and Sample Size (PASS) Software (version 15, 2017). NCSS, LLC. Kaysville, Utah, USA.

A factorial design with two factors (SS and NiTi wires) at 2 and 3 levels has 6 cells (treatment combinations). A total of 66 subjects are required to provide 11 subjects per cell. This design achieves 89% power when an F test is used to test factor of SS wires with a significance level (α) of 0.05 and the effect size is 0.400, achieves 82% power when an F test is used to test factor of NiTi wires at a 5% significance level and the effect size is 0.400, and achieves 82% power when an F test is used to test the SS*NiTi interaction at a 5% significance level and the effect size is 0.400.

**Fig. 1:**
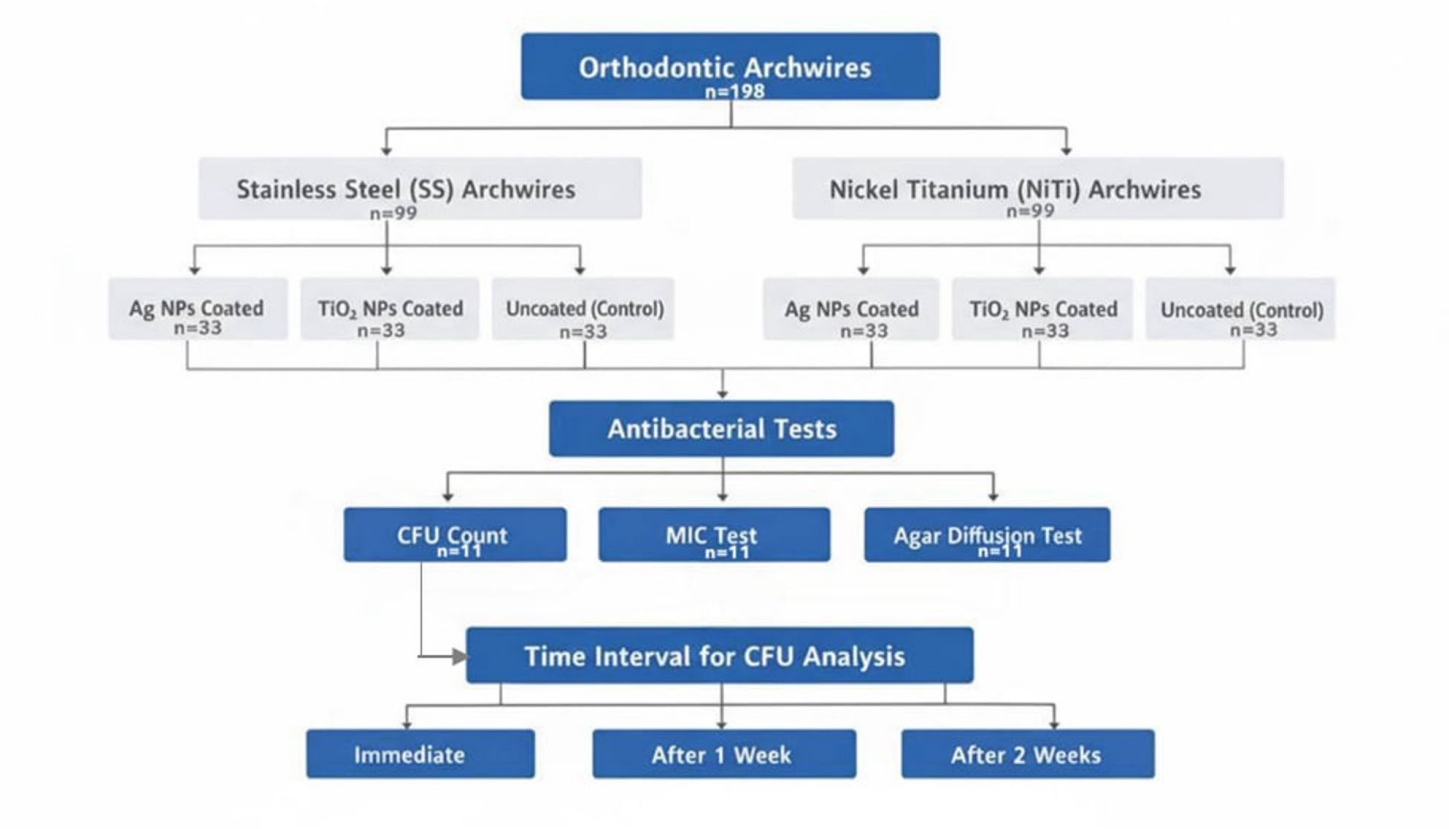
Flow chart of the study diagram

This in vitro study involved 198 arch wires. Set 1 included 99 SS arch wires which were divided into three equal groups (33 uncoated group (control), 33 Ag-NPs coated group and 33 TiO_2_NPs coated group). Each group was subdivided into 3 equal subgroups (11 for CFU and MIC tests, 11 for CFU time intervals test and 11 for agar diffusion zone test). Similarly, set 2 included 99 NiTi arch wires which were divided into three equal groups (33 uncoated group (control), 33 Ag-NPs coated group and 33 TiO_2_NPs coated group). Each group was subdivided into 3 equal subgroups (11 for CFU and MIC tests, 11 for CFU time intervals test and 11 for agar diffusion zone test) (Fig. 1).

### Ag-NPs preparation

Ag-NPs were created at the National Research Center’s laser research unit in Cairo. A high-purity 99.99% Ag target was exposed to radiation using the Nd: YAG nanosecond pulsed laser (PRII 8000 continuum laser; Electro Optics, Inc., Cairo) with the following settings: basic wavelength of 1064 nm, pulse frequency of 10 Hz, pulse width of 6 ns and power of 4 W. The laser beam was focused perpendicularly onto the Ag items using a 10-cm convex lens [17].

### TiO_2_NPs preparation

The TiO_2_NPs were generated at the physics research section of the National Research Center in Cairo by combining prescribed amounts of titanium isopropoxide (TTiP) (76.7 ml) and isopropanol (76.4 ml) into 800 ml of distilled water, which has been adjusted to a pH of 1.5 using nitric acid, hydrochloric acid. A white precipitate was generated upon the addition of TTiP. After two days of agitation at ambient temperature, the precipitate transformed into a clear, yellowish solution. Triethylamine (TEA) was added dropwise to 100 milliliters of the Ti solution until the pH values attained 7, 9, and 11. A teflon container within a SS autoclave was subsequently filled with the resultant white precipitate suspension (150 ml, constituting 75% of the reactor volume). Two distinct temperature and duration conditions were employed for the hydrothermal treatment: 120 °C for 24 h and 150 °C for 6 h. The operating pressures at these temperatures are 475.72 kPa and 198.48 kPa, respectively. Centrifugation was employed to generate TiO_2_NPs, which were subsequently rinsed three times with distilled water. Subsequently, the precipitates were filtered and allowed to dry overnight at 120 °C [18].

### Technique of coating

**Fig. 2:**
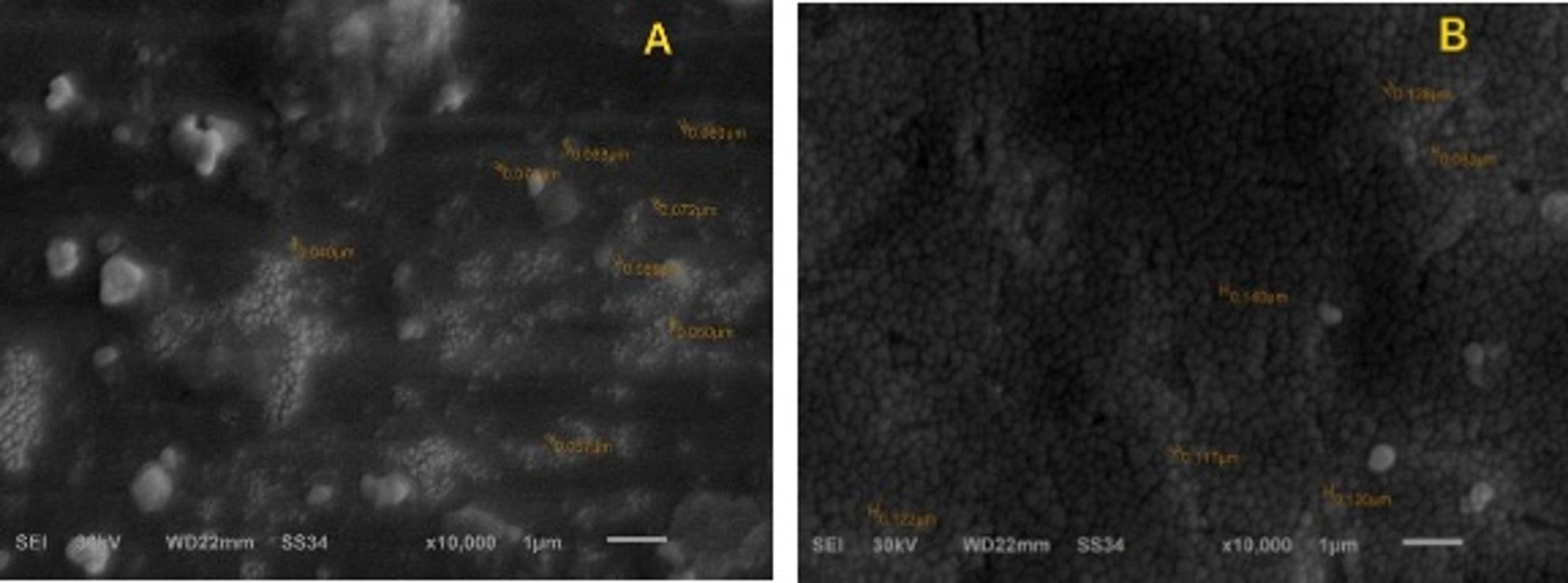
Scanning electron microscope micrographs showing coating thickness on arch wire surfaces. **A** coating thickness on SS arch wire surface. **B** coating thickness on NiTi arch wire surface

Using the sol-gel thin film coating technique, all arch wires were covered by the Ag-NPs and TiO_2_NPs. The arch wires were cleaned using an ultrasonic cleaner set to 50 Hz and 100 watts, with a temperature configuration of 0.05 and an actual temperature of 36 °C. They were kept in 95% ethyl alcohol for 15 min, then in a 0.1 molar sodium hydroxide solution for another 15 min, and finally in two cycles of distilled water, each lasting 15 min. Two rounds of 15-minute distilled water cleaning were performed on the control uncoated arch wires. For half an hour, wires were submerged in plates containing NPs which were prepared at a concentration of 0.1%; each plate could hold eleven wires to prevent contact and ensure complete circumferential exposure testing [19]. The cleaned SS and NiTi arch wires were submerged in the NPs solutions for 30 min. To ensure uniformity and consistent coating thickness, the wires were withdrawn at a constant speed of 10 cm/min. Following the dipping process, the arch wires were air-dried for 5 min and then subjected to thermal curing in a specialized oven at 160 °C for three minutes to facilitate the evaporation of the solvent and the formation of a stable nano-thin film. The resulting coating thickness was approximately 40–140 nm, as verified by scanning electron microscopy (SEM) (Fig. 2). The Ag and TiO_2_ NPs solutions were properly uniformed on the cleaned SS and NiTi arch wires. Finally, the specimens were stored in a dark, desiccated environment to prevent photo-degradation or contamination prior to testing [15, 16, 18–20].

## The electron microscope

**Fig. 3:**
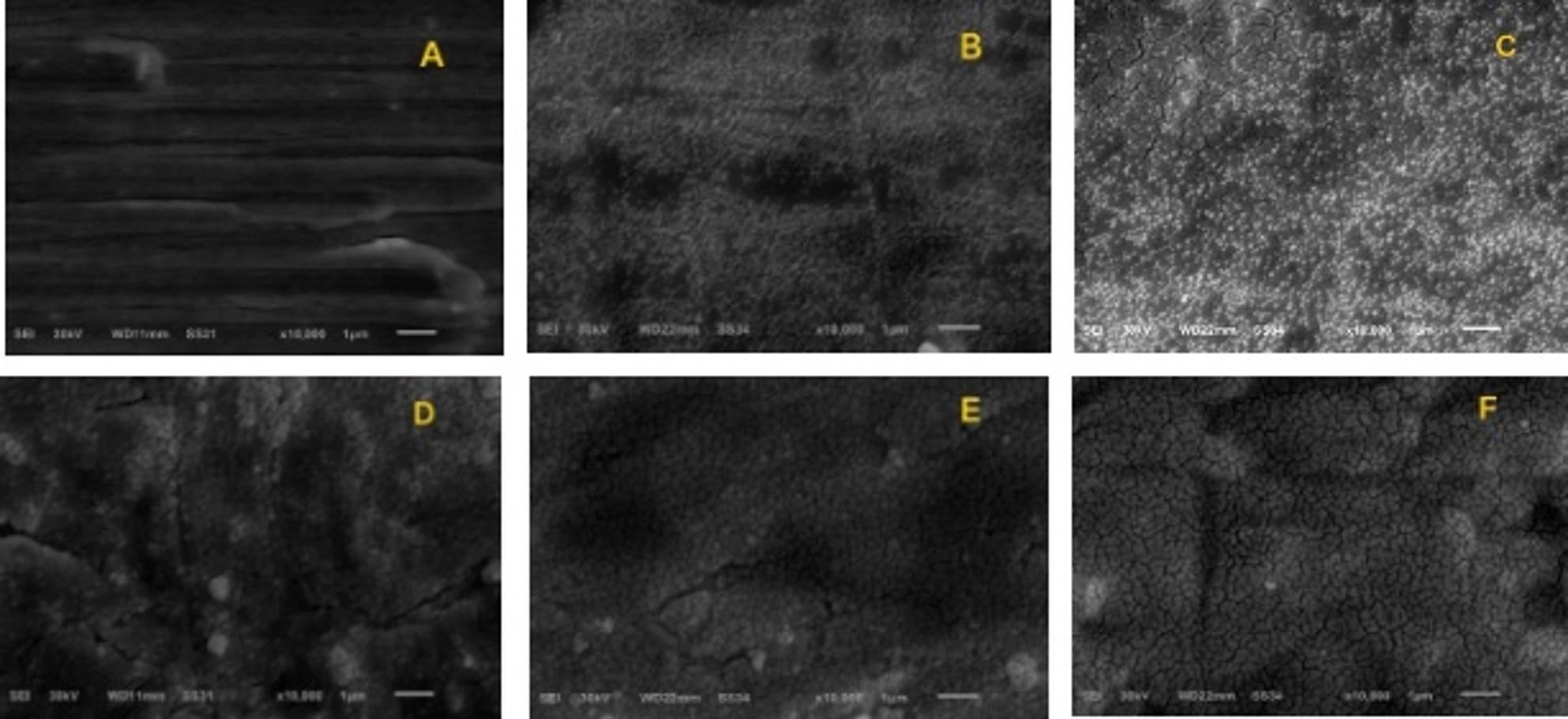
Scanning electron microscope micrographs revealing the layers of coating on arch wire surfaces obtained at a standardized magnification of ×10,000. **A** uncoated SS arch wire surface. **B** SS arch wire coated with Ag-NPs. **C** SS arch wire coated with TiO2NPs. **D** uncoated NiTi arch wire surface. **E** NiTi arch wire coated with Ag-NPs. **F** NiTi arch wire coated with TiO2NPs.

To verify nanocoating, one arch wire was randomly chosen from each group and scanned using an electron microscope (SEM, JSM 6510, LV, Jeol, Akishima, Japan) at magnifications of 1000 ×, 5000 × and 10,000 × (Fig. 3). Samples were ultrasonically cleaned, air-dried and sputter-coated with a thin gold layer prior to SEM examination. Imaging was performed using a scanning electron microscope at an accelerating voltage of 30 kV under high-vacuum conditions [21]. SEM analysis was performed primarily to qualitatively verify NPs deposition on representative samples from each group so, a random arch wire from each group was scanned [22–24].

### Antibacterial tests

#### Antibiofilm activity

##### CFU biofilm

**Fig. 4:**
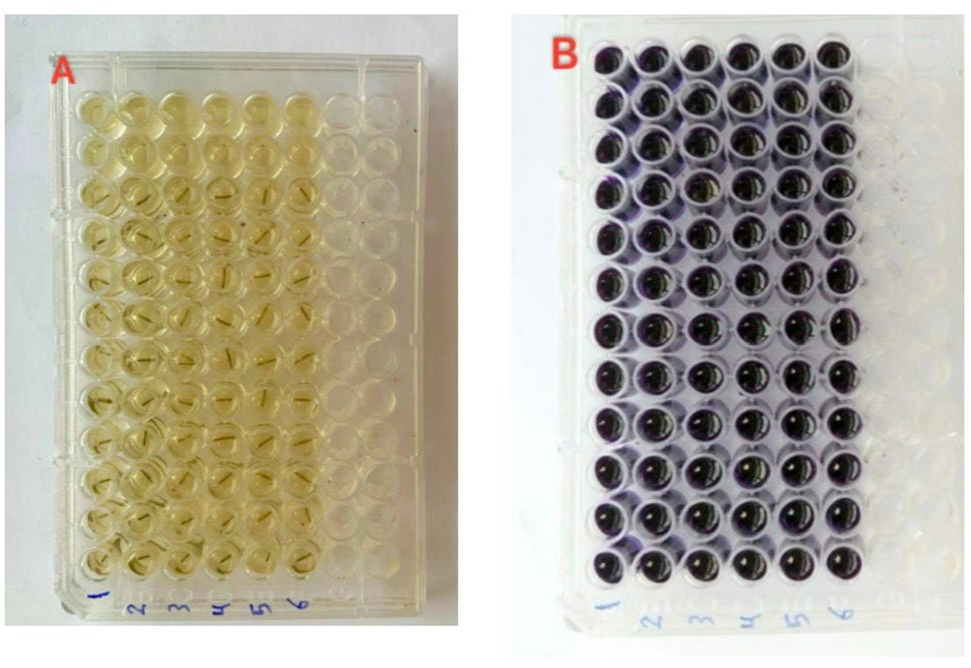
(**A**) Tryptic soy broth (Merck, Germany) injected into 96-well microtiter plates with coated and uncoated wires. **B **Crystal violet solution stain biofilm for MIC biofilm preperation.

**Fig. 5:**
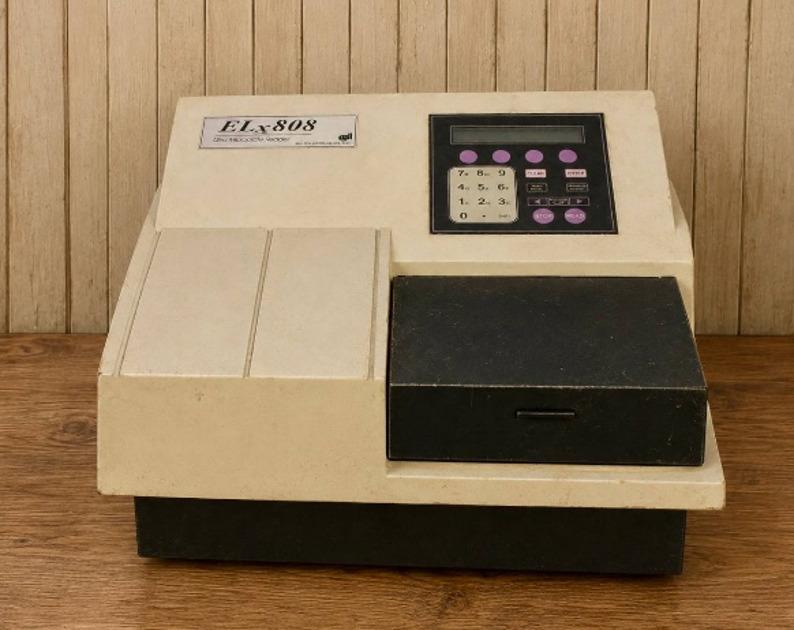
Microplate reader (ELx808™ Absorbance, Biotek, USA)

Coated and uncoated (control) wire pieces measuring 5 mm in length were tested against the reference strain of S. mutans (ATCC 25175) for biofilm prevention. Sterile 10 ml tryptic soy broth (Merck, Germany) was used to grow bacterial isolates. A 0.5 McFarland standard (1.5 × 10^5^ CFU/ml) bacterial inoculum was injected into 96-well microtiter plates with coated and uncoated wire pieces (Fig.4 A). After 24 h at 37 °C, the absorbance was quantified at 490 nm using a microplate reader (ELx808™ Absorbance, Biotek, USA) (Fig. 5) to read the bacterial colony count for CFU assay [25, 26]. 

##### CFU time intervals biofilm

Using Fusayama’s saliva composition with a pH of 6.75 consisted of NaCl (0.4 g/L), KCl (0.4 g/L), CaCl_2_.2H_2_O (0.795 g/L), NaH_2_PO_4_ (0.69 g/L), Na_2_S.9H_2_O (0.005 g/L), urea (1 g/L) and distilled water up to 1 L for incubation of the tested SS and NiTi arch wires for the CFU time intervals test. Samples were incubated at 37 °C to simulate oral conditions [27, 28]. The CFU test was conducted after incubation of all samples in 10 ml of the artificial saliva with time intervals of (immediately, after one week and after 2 weeks) to investigate the duration of the antibacterial effect of the nano-coated wires as the ‘immediate’ time point refers to measurements performed directly after NPs coating, serving as a baseline reference for comparison with samples subjected to incubation in artificial saliva periods simulating oral environmental conditions [29].

##### MIC biofilm

The reference strain of S. mutans (ATCC 25175) was used to evaluate coated and uncoated (control) wire sections measuring 5 mm in length for biofilm prevention. Bacterial isolates were grown in sterile 10 ml tryptic soy broth (Merck, Germany). Coated and uncoated wire segments were inserted into 96-well microtiter plates containing a 0.5 McFarland standard (1.5 × 10^5^ CFU/ml) bacterial inoculum. The plates were washed and allowed to air dry at room temperature for the MIC confirmation test after a 24-hour period at 37 °C. Two hundred microliters of 0.1% w/v crystal violet solution were applied and incubated for 30 min before rinsing and drying (Fig. 4B). Subsequently, 100 µl of 96% ethanol was used to remove the stained bound biofilm. Similarly, the absorbance was quantified at 490 nm using a microplate reader (ELx808™ Absorbance, Biotek, USA). where lower optical density values indicated greater antibacterial activity and biofilm inhibition [25, 26, 30]. The reduction of biofilm was quantified and compared to the control group.

### Antibacterial activity (agar diffusion test)

**Fig. 6:**
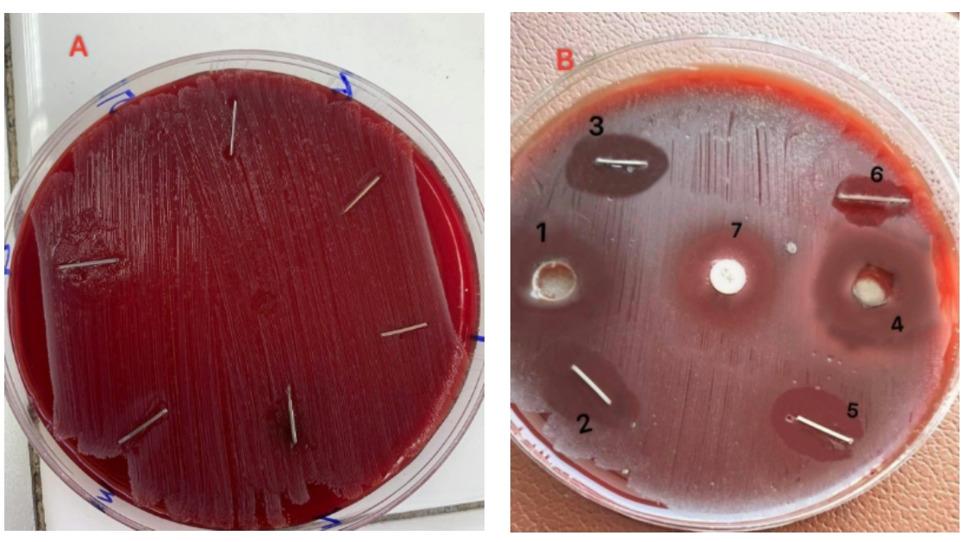
(**A**) The disc diffusion of control groups. **B** The disc diffusion of (1) Ag-NPs solution, (2) Ag-NPs coated NiTi arch wire, (3) Ag-NPs coated SS arch wire, (4) TiO_2_NPs solution, (5) TiO_2_NPs coated SS arch wire, (6) TiO_2_NPs coated NiTi arch wire, (7) antibiotic disc

The standard S. mutans strain was stored at − 70 °C and spectrophotometer-prepared at 0.5 McFarland standards. Each blood agar plate of the total 11 agar plates received a 50 µ L sample of each dilution. The tested SS and NiTi arch wires were UV sterilized. Agar plates were uniformly inoculated for coated and uncoated arch wires insertion using sterile spreaders. From each batch, 10 mms arch wire segments were randomly selected from every group. The groups had sterile holders for implanting arch wire segments into agar plates. Under minimal pressure, the wire was carefully preserved in the liquid and incubated at 37 °C for 48 h. The clearing zone diameter was measured using a digital caliper after 48 h [31] An antibiotic disc was additionally incorporated as a positive control in the agar diffusion assay to validate bacterial growth inhibition and to provide a reference standard for comparison of the antibacterial activity of both NPs solutions and the tested NPs-coated arch wires (Fig. 6).

### Statistical analysis test

Data was entered and evaluated using IBM-SPSS software (IBM Corp. Released 2020). GraphPad Prism 9.5.1 for Windows and IBM SPSS Statistics for Windows, Version 27.0, Armonk, NY: IBM Corp were utilized., The normality of the quantitative data was first checked with Q-Q plots and Shapiro-Wilk’s test (*p* > .050 indicates normally distributed data). Significant outliers were checked for in the boxplots. Mean and standard deviation (SD) or standard error (SE) were used to express quantitative data. To find out if the two independent factors (coating NPs and arch wire) interact with the continuous dependent variable as CFU test, we utilized the two-way ANOVA test. The primary effects of the arch wire and coated NPs were then reported, since the interaction effect had no statistical significance. A statistically significant difference was quantified using partial eta squared (η2). If the partial eta squared (η2) was 0.01, 0.06, or 0.14, the effect size was categorized as small, medium, or large, respectively. When the p-value was less than or equal to.050, the results were deemed statistically significant for all tests.

For the ion release test. To comprehend the impact of wire type, NPs type, and time on the parameters under study, a three-way mixed ANOVA was conducted. Using Levene tests, we checked for variance homogeneity (*p* > .05). A two-way interaction between the wire and NPs was conducted with respect to time in order to achieve a three-way interaction. At each time point, a two-way interaction between the wire and NPs was conducted in order to achieve a three-way interaction.

Statistical analysis was structured to evaluate the effects of NPs coating of SS and NiTi arch wires on S mutans bacteria across agar diffusion, MIC and CFU at different time intervals. Repeated measures analysis was applied for time-dependent data, while subgroup comparisons were performed using appropriate post hoc tests. The analysis was simplified to emphasize clinically relevant comparisons and improve interpretability.

## Results

### Antibiofilm activity

#### CFU biofilm

At the immediate time interval, no statistically significant difference was observed between SS and NiTi arch wires (*p* = .671; small effect size). In contrast, NPs coating demonstrated a statistically significant effect on CFU counts (*p* = .007; large effect size), with higher bacterial counts observed in the control group compared to both Ag-NPs and TiO_2_NPs groups. No significant interaction was found between arch wire type and NPs coating (*p* = .953) (Table 1).

**Table 1:** Evaluation of CFU Biofilm in control, Ag-NPs and TiO_2_NPs coated SS and NiTi arch wires

Arch wire	Nanoparticles	Mean	SD	F (2, 60)	Sig.	Partial η^2^
SS	Control	0.07491	0.010977	0.048	0.953	0.002
	Ag	0.06164	0.016077			
	TiO_2_	0.06345	0.018806			
NiTi	Control	0.07627	0.013654			
	Ag	0.06191	0.014943			
	TiO_2_	0.06645	0.012445			
Main effect of arch wires on the CFU Biofilm.						

Arch wire	**N**	**Mean**	**SE**	**F (1**,** 60)**	**Sig.**	**Partial η** ^**2**^
SS	33	0.067	0.003	0.182	0.671	0.003
NITi	33	0.068	0.003			
Main effect of coating nanoparticles on the CFU Biofilm.						

Nanoparticles	**N**	**Mean**	**SE**	**F (2**,** 60)**	**Sig.**	**Partial η** ^**2**^
Control	11	0.076	0.003	5.329	0.007	0.151
Ag-NPs	11	0.062	0.003			
TiO_2_NPs	11	0.065	0.003			

Notes: *SD *standard deviation, *Sig* statistical significance (p-value). Partial eta squared (η^2^) is a measure of effect size. F (2, 60) = F-statistic at 2 and 60 degrees of freedom. F (1, 60) = F-statistic at 1 and 60 degrees of freedom. The test of significance is two-way ANOVA

### CFU time intervals biofilm

**Fig. 7:**
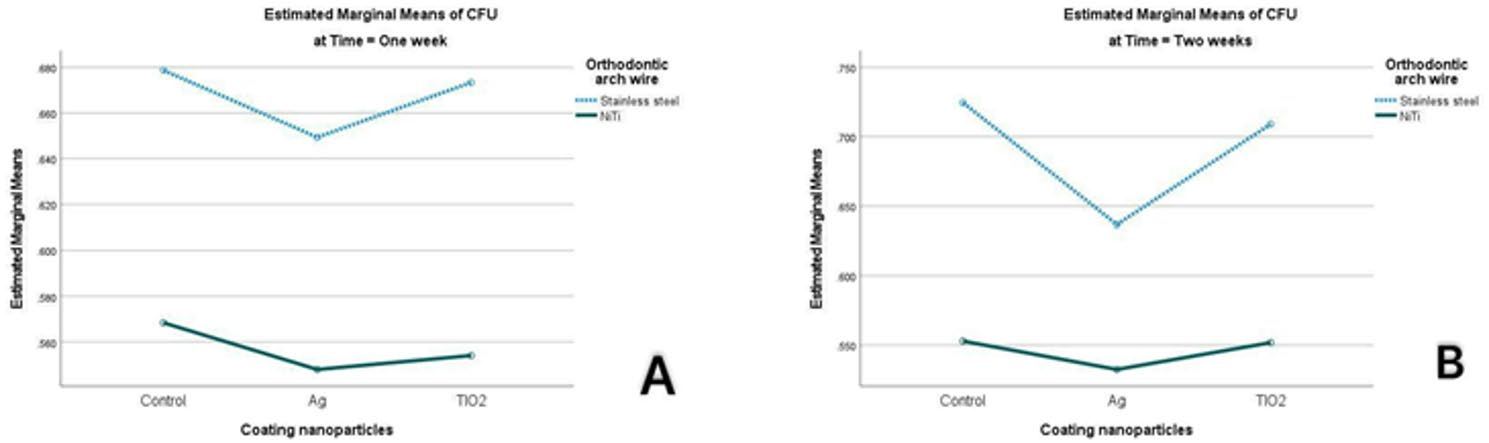
**A** Antibacterial effect (CFU Biofilm) in different nanoparticles after one week of incubation in artificial saliva. **B** Antibacterial effect (CFU Biofilm) in different nanoparticles after two weeks of incubation in artificial saliva

Time-dependent analysis revealed no significant three-way interaction among arch wire type, NPs coating and time (*p* = .933). As comparison of mean CFU biofilm at different time intervals of immediately, after 1 week and after 2 weeks revealed insignificant three-way interaction between orthodontic arch wire type, coating NPs type and time [F (2.5, 74.2) = 0.106, partial eta-squared (η2) = 0.004, *p* = .933] using Greenhouse-Geisser method as sphericity was not assumed (*p* = .001) and Epsilon was 0.618 (i.e., < 0.750). Therefore, two-way interaction procedures were performed to assess the effect of orthodontic arch wire and time, as well as coating NPs and time on CFU biofilm. Regarding orthodontic arch wire type and time, there was no statistically significant two-way interaction effect [F (1.3, 82.5) = 3.583, partial eta-squared (η2) = 0.053, *p* = .051] using Greenhouse-Geisser method as sphericity was not assumed (*p* < .001) and Epsilon was 0.645 (i.e., < 0.750). Regarding coating NPs type and time, there was no statistically significant two-way interaction effect [F (2.5, 80.1) = 0.992, partial eta-squared (η2) = 0.031, *p* = .391] using Greenhouse-Geisser method as sphericity was not assumed (*p* < .001) and Epsilon was 0.636 (i.e., < 0.750) (Fig. 7).

Overall, NPs coatings significantly reduced CFU counts compared with the control.

### MIC biofilm

There was a statistically insignificant main effect of arch wire type on MIC biofilm with small effect size (*p* = .583). The total antibacterial activity of the NPs varied significantly (*p* = .007). The concentration was higher in control > TiO_2_NPs > Ag-NPs with large effect size. The interaction between the type of coating materials and arch wire materials was insignificant (*p* = .931), with small effect size (Table 2). These findings indicate that NPs coatings, particularly Ag-NPs, significantly enhance antibacterial efficacy.

**Table 2:** Evaluation of MIC Biofilm in control, Ag-NPs and TiO_2_NPs coated SS and NiTi arch wires

Arch wire	Nanoparticles	Mean	SD	F (2, 60)	Sig.	Partial η^2^
SS	Control	0.60500	0.168524	0.071	0.931	0.002
	Ag	0.45827	0.131289			
	TiO_2_	0.51682	0.140218			
NiTi	Control	0.61700	0.224299			
	Ag	0.46809	0.110667			
	TiO_2_	0.55827	0.129843			
Main effect of arch wires on the MIC Biofilm.						

Arch wire	**N**	**Mean**	**SE**	**F (1**,** 60)**	**Sig.**	**Partial η** ^**2**^
SS	33	0.527	0.027	0.304	0.583	0.005
NITi	33	0.548	0.027			
Main effect of coating nanoparticles on the MIC Biofilm.						

Nanoparticles	**N**	**Mean**	**SE**	**F (2**,** 60)**	**Sig.**	**Partial η** ^**2**^
Control	11	0.611	0.033	4.983	0.010	0.142
Ag-NPs	11	0.463	0.033			
TiO_2_NPs	11	0.538	0.033			

Notes: SD  standard deviation, Sig statistical significance (p-value), Partial eta squared (η^2^) is a measure of effect size. F (2, 60)  F-statistic at 2 and 60 degrees of freedom. F (1, 60)  F-statistic at 1 and 60 degrees of freedom. The test of significance is two-way ANOVA

### Antibacterial activity (agar diffusion test)

 There was a significant difference in antibacterial activity between arch wires (*p* = .008) as it was higher in SS than NiTi wires with medium effect size. The total antibacterial activity of the NPs varied significantly (*p* < .001) as it was higher in Ag-NPs > TiO_2_NPs > control with large effect size. The interaction between type of coating materials and arch wire materials was insignificant (*p* = .137), with medium effect size (Table 3). Negative or near-zero values observed in agar diffusion measurements were adjusted to zero, indicating absence of detectable inhibition zones rather than true negative activity. Overall, Ag-NPs demonstrated the highest diffusion-based antibacterial activity, followed by TiO_2_NPs, with consistent trends across both arch wire types.

**Table 3:** Evaluation of agar diffusion zone effect in control, Ag-NPs and TiO_2_NPs coated SS and NiTi arch wires

Arch wire	Nanoparticles	Mean	SD	F (2, 60)	Sig.	Partial η^2^
SS	Control	0.00	0.000	2.058	0.137	0.064
	Ag	12.73	4.174			
	TiO_2_	9.27	2.901			
NiTi	Control	0.00	0.000			
	Ag	9.91	2.023			
	TiO_2_	7.36	1.804			
Main effect of arch wires on the agar diffusion zone effect.						

Arch wire	**N**	**Mean**	**SE**	**F (1**,** 60)**	**Sig.**	**Partial η** ^**2**^
SS	33	7.333	0.409	7.408	**0.008**	0.110
NITi	33	5.758	0.409			
Main effect of coating nanoparticles on the agar diffusion zone effect.						

Nanoparticles	**N**	**Mean**	**SE**	**F (2**,** 60)**	**Sig.**	**Partial η** ^**2**^
Control	11	-2.665^− 15^	0.501	136.775	**< 0.001**	0.820
Ag-NPs	11	11.318	0.501			
TiO_2_NPs	11	8.318	0.501			

Notes: SD  standard deviation, Sig statistical significance (p-value). Partial eta squared (η^2^) is a measure of effect size. F (2, 60)  F-statistic at 2 and 60 degrees of freedom. F (1, 60)  F-statistic at 1 and 60 degrees of freedom. The test of significance is two-way ANOVA

## Discussion

This investigation evaluates the degree of bacterial colonization on Ag-NPs and TiO_2_NPs coated SS and NiTi wires against S. mutans bacterium as a trial to improve the issue of microbial plaque formation on orthodontic arch wires. A modern nanotechnology was applied to orthodontic arch wires to assess the influence of Ag-NPs and TiO_2_NPs coatings on SS and NiTi arch wires using CFU counts, MIC determination, agar diffusion tests and time-dependent analysis. The findings demonstrate that NPs coatings significantly influence antibacterial behavior; however, their effectiveness is dependent on multiple interacting factors, including NPs type, time and substrate material. The significant reduction in bacterial viability observed with both Ag-NPs and TiO_2_NPs coatings confirms that surface modification of orthodontic materials can effectively limit bacterial colonization. Rather than merely demonstrating antibacterial activity, the present findings highlight differences in magnitude and persistence, with Ag-NPs consistently exhibiting stronger antibacterial effects across all assays. This superior performance may be attributed to their well-documented ability to disrupt bacterial cell membranes, generate reactive oxygen species, and release bioactive ions, thereby exerting both contact-dependent and sustained antimicrobial effects [10–12]. In contrast, although TiO_2_NPs demonstrated significant antibacterial activity, their comparatively lower efficacy may be related to differences in antibacterial mechanisms, which are often dependent on photocatalytic activation [13, 14].

NiTi and SS rectangular arch wires of 0.017 × 0.025 inches were employed in this study. The NiTi alloy is the most effective shape memory alloy providing orthodontic forces that influence tooth movement, particularly in the first stages of orthodontic therapy [32]. The SS arch wires are viable during the initial and finishing phases of therapy due to their high mechanical properties [33]. The comparison between SS and NiTi arch wires revealed differences in antibacterial performance, with SS generally demonstrating greater efficacy. While this may be partially attributed to differences in surface characteristics such as roughness and coating uniformity, this explanation remains hypothetical [34], as surface topography was not directly measured in the present study. It is well recognized that bacterial adhesion is influenced by multiple factors, including surface energy, chemical composition and salivary pellicle formation [35]. Therefore, the observed alloy-dependent differences should be interpreted with caution and require further investigation. The sol-gel coating method was chosen for this investigation because of its improved porosity control, high precursor ingredient purity and excellent coating layer homogeneity providing stable coatings with favorable surface characteristics, which are essential for consistent antibacterial performance [15]. Electron microscopy scanning analysis in the present study confirmed successful NPs deposition on both SS and NiTi wire surfaces, supporting the validity of the coating protocol. In addition to statistical significance, effect size analysis demonstrated that the observed differences between NPs coatings were of moderate to large magnitude, indicating potential practical relevance despite the in vitro nature of the study as observed statistical effect sizes suggest that for example NPs coatings may have a clinically meaningful influence on antibacterial behavior facilitating more WSLs preventions leading to good oral hygiene and less post orthodontic treatment caries [36, 37].

CFU is a unit used to estimate the number of viable microorganisms in the media [38]. The CFU results demonstrated a significant reduction in bacterial biofilm formation on Ag-NPs and TiO_2_NPs coated wires compared with uncoated controls nearly at the same level. This finding supports the concept that surface modification of orthodontic materials can effectively limit bacterial adhesion and proliferation [39]. The strong antibacterial effect observed in the present study further confirms the suitability of Ag-NP and TiO_2_NPs coatings for orthodontic applications aimed at reducing cariogenic bacterial load although TiO_2_NPs had a lesser extent than Ag-NPs. However, TiO_2_NPs are characterized by chemical stability and favorable biocompatibility, making them an attractive alternative for surface modification of orthodontic materials [13]. The CFU findings are in agreement with Oves et al. [40] who confirmed that Ag-NPs exhibit strong bactericidal activity by disrupting bacterial cell membranes and inhibiting metabolic processes, leading to marked reductions in viable bacterial counts. In contrast, Ahn et al. [41] reported that bacterial adhesion may be more strongly influenced by surface roughness and alloy composition than by antibacterial surface modification alone, resulting in inconsistent CFU reduction across different orthodontic materials.

The time-dependent analysis of CFU revealed a gradual decline in antibacterial activity following incubation in artificial saliva, with a marked reduction after one and two weeks. Ag-NPs coated wires retained detectable antibacterial effects after one week especially in Ag-NPs coated SS wires, the activity of all coatings was substantially diminished after two weeks. The time-dependent reduction in antibacterial activity observed in this study represents a clinically relevant finding. Although both coatings demonstrated strong initial antibacterial effects, a gradual decline was observed following incubation in artificial saliva. This reduction may be related to NPs depletion, surface passivation or NPs ion release over time. However, as no direct measurements of coating degradation or ion release kinetics were performed, these mechanisms remain speculative. This limitation highlights the need for future studies incorporating physicochemical characterization to better understand the durability of NPs coatings under simulated oral conditions. These findings are consistent with the study of Kumar et al. [42] who reported that NP coatings may lose antibacterial effectiveness due to ion depletion, coating degradation, or surface saturation under prolonged exposure to oral conditions. In contrast, Li et al. [43] who demonstrated that the reduced durability observed in their study may therefore be related to the single-layer coating approach employed.

MIC test was used in this investigation as a confirmatory step for the CFU test results against S. mutans. MIC” is the lowest concentration of an antimicrobial agent (such as an antibiotic) that inhibits the visible growth of a bacterium after overnight incubation” as the high MIC means the material is less effective at eradicating bacteria and low MIC means the material has more powerful antibacterial effect [44].The MIC results indicated that Ag-NPs exhibited lower inhibitory concentrations against S. mutans than TiO_2_NPs, reflecting that Ag-NPs had stronger antibacterial potency. This observation is consistent with the findings of Kim et al. [45] who demonstrated that Ag-NPs possess low MIC values against oral bacteria. Yin et al. [46] similarly reported superior antibacterial efficiency of Ag-NPs compared with metal oxide NPs, attributing this effect to their high surface reactivity and sustained ion release. However, Allaker [47] highlighted that MIC values of NPs may vary considerably depending on particle size, synthesis method, and surface functionalization. Such methodological variability may account for discrepancies in MIC values reported across different studies and emphasizes the importance of standardized NPs preparation protocols.

The short-term antibacterial effectiveness observed in this study may be clinically beneficial during the early stages of orthodontic treatment, when plaque accumulation and enamel demineralization risk are highest.

The agar diffusion test (the minimum zone of inhibition) is a quick and an easy test. The agar diffusion test revealed larger zones of inhibition around Ag-NPs coated wires compared with TiO_2_NPs coated, further supporting the superior antibacterial diffusion-based activity of Ag-NPs. These findings are consistent with those of Espinosa-Cristóbal et al. [10], who reported pronounced inhibition zones surrounding Ag-NPs modified orthodontic appliances. Similarly, However, it should be noted that the agar diffusion method relies on the diffusion of antimicrobial agents into the agar medium and may underestimate the antibacterial efficacy of surface-bound NPs. This limitation may explain discrepancies reported by Sodagar et al. [48] who observed limited inhibition zones despite confirmed antibacterial activity using CFU based methods. The coating on SS wires had higher antibacterial effect than NiTi wires. As a comparative study of the surface topography of NiTi and SS arch wires showed that the NiTi arch wires had a higher level of roughness [49]. A strong positive relationship is shown between elevated surface roughness of orthodontic wires and augmented bacterial colonization particularly S. mutans levels as rougher uneven surfaces offer greater surface area and sheltered places for bacterial adherence [50]. So, SS arch wires exhibited significantly greater antibacterial efficacy than NiTi arch wires in all nano-coated groups. This finding is consistent with Amini [51] and Yu et al. [52] who reported higher surface roughness and greater bacterial adhesion on NiTi wires compared with SS ones. The smoother surface of SS wires may facilitate more uniform NPs deposition and enhance antibacterial effectiveness. However, Eliades and Bourauel [53] reported no significant difference in bacterial adhesion between SS and NiTi alloys when exposed to salivary proteins, suggesting that biofilm formation is a multifactorial process influenced by surface chemistry and oral environmental factors. These findings indicate that alloy-dependent antibacterial behavior may vary under different experimental and clinical conditions.

The use of CFU, MIC and agar diffusion assays in combination provides a comprehensive evaluation of antibacterial activity through complementary mechanisms. Different wire lengths were selected according to the methodological requirements of each microbiological assay. 5 millimeters segments were used for CFU and MIC assays to allow standardized immersion within microtiter wells and ensure uniform bacterial exposure, whereas 10 millimeters segments were used for agar diffusion testing to facilitate stable placement within agar media and allow accurate measurement of inhibition zones. CFU analysis reflects the direct bactericidal effect by quantifying viable bacterial colonies following exposure to the tested materials. In contrast, MIC determination identifies the minimum concentration required to inhibit visible bacterial growth, representing the intrinsic antimicrobial potency of the NPs. Agar diffusion testing evaluates the ability of antimicrobial agents to diffuse from the coated surfaces into the surrounding medium, thereby reflecting diffusion-mediated antibacterial activity [31, 38, 44]. The integration of these methods allows differentiation between contact-dependent antibacterial effects and diffusion-based inhibition, providing a more comprehensive understanding of the antibacterial performance of NPs coatings. This multi-assay approach enhances the reliability of the findings and supports a more robust interpretation of the observed antibacterial behavior.

While the superior antibacterial activity of Ag-NPs has been widely reported, the present study provides additional insight by quantifying the magnitude and temporal persistence of this effect in comparison with TiO_2_NPs. Across all assessment methods, Ag-NPs demonstrated consistently greater antibacterial efficacy, reflected not only in statistically significant differences but also in moderate-to-large effect sizes, indicating meaningful practical relevance. Importantly, the differences between coatings were not limited to initial performance but extended to time-dependent behavior, where Ag-NPs maintained detectable antibacterial activity for a longer duration compared with TiO_2_NPs. The significant reduction in bacterial viability observed with Ag-NPs and TiO_2_NPs coatings in this study confirmed that the integration of nanotechnology significantly improves the clinical performance of dental materials. Ahuja et al. [54] demonstrated that Ag-NPs possess potent broad-spectrum antibacterial activity, particularly against *S. mutans*, by disrupting bacterial cell walls and inhibiting DNA replication. This aligns with our findings where Ag-NPs consistently exhibited the highest antibacterial activity across all tests, likely due to the sustained release of bioactive ions.

This suggests that, beyond intrinsic potency, the stability and sustained activity of the coating are critical determinants of clinical usefulness. From a clinical perspective, even modest differences in antibacterial effectiveness may be relevant during the early stages of orthodontic treatment, when plaque accumulation and enamel demineralization risk are highest therefore, the findings of this study highlight that the comparative value of nanoparticle coatings lies not only in their antibacterial capability, but also in their effect size, durability, and consistency across different conditions, which are essential considerations for potential clinical translation [8, 46].

Furthermore, while our study focused on the antibacterial properties of arch wire coatings, it is essential to consider the impact of such modifications on the overall mechanical stability of the orthodontic system suggesting that nano-coatings not only serve an antimicrobial purpose but may also contribute to the physical durability of the appliance during treatment and also can improve the chemical properties of the dental materials as Ahuja et al. [55] reported that NPs surface coatings on SS brackets can actually improve biocompatibility by acting as a protective barrier that reduces the leaching of toxic metal ions like Ni and Chromium, thereby decreasing cytotoxicity and genotoxicity in oral mucosal cells. In addition to Elsonny et al. [20] who reported that Ag-NPs and TiO_2_NPs coatings on SS and NiTi orthodontic arch wires may reduce ion release and improve surface characteristics under simulated oral conditions. These combined findings support the use of NPs coatings as a multi-functional strategy to improve the orthodontic materials properties.

### Limitations

The present study has several limitations that should be considered when interpreting the findings. First, the in vitro design does not fully replicate the complex biological, chemical, and mechanical conditions of the oral environment, including salivary flow, pH fluctuations, dietary influences and host immune responses. The antibacterial evaluation was performed using a single bacterial strain (S. mutans), whereas oral biofilms are polymicrobial and involve complex microbial interactions that may influence adhesion and antibacterial efficacy. In addition, potential changes in coating integrity over time, including degradation or ion release dynamics, were not directly measured. The observed time-dependent reduction in antibacterial activity may be related to NPs depletion or coating degradation; however, these mechanisms remain speculative, as no direct measurements of ion release kinetics or coating integrity were performed. Surface characteristics such as roughness and wettability, which may influence bacterial adhesion, were not evaluated in the present study. Furthermore, biocompatibility and cytotoxicity of the NPs coatings were not directly investigated in the present study. However, the NPs concentrations used were selected based on previously published clinical studies reporting acceptable biological safety profiles [56–58]. Moreover, SEM analysis was limited to a single representative specimen per group, which may not fully reflect coating uniformity across all samples. The reduction in antibacterial activity over time may be related to NPs depletion or coating degradation; however, this remains speculative as no direct measurements of coating thickness or surface topography after incubation were performed. The reduction in *S. mutans* levels may indicate a possible role of NPs coatings in modulating factors associated with enamel demineralization; however, the development of WSLs is a multifactorial process influenced by dietary habits, oral hygiene practices, salivary composition and host-related factors [2, 47]. Consequently, further in vivo and clinical studies are required to determine whether the observed antibacterial effects translate into measurable reductions in caries incidence or WSL formation.

### Recommendations

Within the limitations of the study, future studies should address the following:

1. Evaluate NPs coatings using polymicrobial biofilm models.
2. Assess long-term coating durability and NPs release kinetics.
3. Quantitatively evaluate coating roughness and wettability.
4. Investigate cytotoxicity and biocompatibility before clinical application.
5. Conduct in vivo and clinical studies to validate antibacterial performance under oral conditions.

## Conclusion

This in vitro investigation demonstrated that coating SS and NiTi orthodontic arch wires with Ag-NPs and TiO_2_NPs significantly reduced *S. mutans* growth and biofilm formation compared with uncoated wires. In the CFU assay, both NPs coatings demonstrated nearly comparable antibacterial effects, whereas Ag-NPs exhibited greater antibacterial activity than TiO_2_NPs in MIC and agar diffusion assays. Although antibacterial effectiveness gradually decreased over time following immersion in artificial saliva, both NPs coatings maintained promising short-term antibacterial properties. These findings suggest that NPs surface modification of orthodontic arch wires may contribute to reducing bacterial colonization during orthodontic treatment.

## Data Availability

All datasets used and analyzed during the current study are available from the corresponding author on reasonable request.

## Abbreviations

NPs: Nanoparticles
Ag: Silver
Ag-NPs: Silver nanoparticles
Ni: Nickel
Ti: Titanium
TiO_2_: Titanium dioxide
TiO_2_NPs: Titanium dioxide nanoparticles
TEA: Triethylamine
TTiP: Titanium isopropoxide
NiTi: Nickel-Titanium alloy
SS: Stainless-steel
SEM: Scanning Electron Microscope
WSLs: White spot lesions
S. mutans: Streptococcus mutans
ANOVA: Analysis of variance
η²: Partial eta squared
F: F-statistic
SD: Standard deviation
SE: Standard error
IBM-SPSS: International Business Machines Statistical Package for the Social Sciences
UV: Ultraviolet
pH: Potential of hydrogen
ATCC: American Type Culture Collection
CFU: Colony forming unit
McF: McFarland standard
MIC: Minimum inhibitory concentration
